# Supplementary material for: Effects of aerobic exercise and resistance exercise on physical indexes and cardiovascular risk factors in obese and overweight school-age children: A systematic review and meta-analysis
Source: PLoS One. 2021 Sep 20;16(9):e0257150. doi: 10.1371/journal.pone.0257150 (PMC8452003; doi:10.1371/journal.pone.0257150)
Supplement: Attachment — Submitted filename: Response to Reviewers.docx [file pone.0257150.s004.docx]

Replies to the reviewers’ comments:

Reviewer #1:

1. pay attention to minor grammar signs (i.e. the comma between VO2peak and triglycerides in abstract section; the arrow in Figure 1)

Response: We feel sorry for our carelessness. In our resubmitted manuscript, those mistakes are polished. Thanks for your correction.

2. in results section of abstract, try to be more coincise and not to write all those numbers in brackets: so, your results will shine

Response: Based on your comments, we have made the optimization to make the unit harmonized within the abstract

.

3. in introduction, I suggest the following reference (https://link.springer.com/article/10.1007/s40292-019-00352-2): not only child are obese, but almost half of their parents classified their weight status incorrectly!

Response: We sincerely appreciate the valuable comments. We have checked the literature carefully, and we considered the most suitable position, and we added references on [49] into the DISCUSSION part in the revised manuscript.

4. in discussion, I suggest the following reference (https://pubmed.ncbi.nlm.nih.gov/32512767/), to stress the concept of the benefits of sport in several disease, such as obesity: not everyone know that sport is so good!

Response: We have also checked the literature carefully, and we added references on [59] into the DISCUSSION part in the revised manuscript.

Reviewer #2:

1. The English language needs to be improved.

Response: Thank you for your valuable and thoughtful comments. We have asked Dr HOU who is a high-level professor carefully checked and polised tour manuscript. Please see if the revised version met the English presentation standard.

2. Abstract: a short background clarifying the importance of the study question should be demonstrated.

Response: We think this is an important suggestion. We have added the background into the Abstract part.

3. Conclusion: This section does not demonstrate new information. It is known that both AE and RE improve these parameters. The authors have to compare between the two types of exercise.

Response: Thank you for pointing this out. Although we agree that this is an important consideration, there are only two articles related to resistance exercise in children with obesity and overweight at school age, which is far less than the number of 12 articles about aerobic exercise, so we cannot compare the effects of the two types of exercises. Moreover, we added this reason to Conclusion part to help readers better understand.

4. Introduction: The authors should focus on the novelty and importance of the study in this section. Discuss, what is the difference between this study and other previous studies.

Response: Thank you! We have added the suggested content to the manuscript on Lines 127-129.

5. Methods: How the data were analyzed in detail?

Response: We have written the process of data analysis in detail in the Statistical Analysis part. Since meta-analysis is implemented with the software of Revman, we input the data into the software, and the software will automatically generate a forest plot to show our data, and the forest plots are also presented in the result part. Thank you for your question.

6. Conclusion: In the conclusion section pleases highlight better the scientific/clinical relevance of your work. Please provide a clear “research message” of the importance of this paper in the scientific community.

Response: Thank you! We have added the suggested content to the manuscript on Lines 409-413.

Reviewer #3:

1. Figure 2 needs to be explained more about colours and signs.

Response: We feel sorry for our carelessness. We have added the suggested content to the manuscript on Lines 243-249.

2. in page 14 in the first section (study selection and Eligibility criteria) last sentence is incomplete.

Response: Thank you for pointing this out. We have polished this part.

Once again, thank you very much for your constructive comments and suggestions which would help us to improve the quality of the paper.
